# Human Stem Cell-like Memory T Cells Are Maintained in a State of Dynamic Flux

**DOI:** 10.1016/j.celrep.2016.11.037

**Published:** 2016-12-13

**Authors:** Raya Ahmed, Laureline Roger, Pedro Costa del Amo, Kelly L. Miners, Rhiannon E. Jones, Lies Boelen, Tinhinane Fali, Marjet Elemans, Yan Zhang, Victor Appay, Duncan M. Baird, Becca Asquith, David A. Price, Derek C. Macallan, Kristin Ladell

**Affiliations:** 1 Institute for Infection and Immunity, St. George’s, University of London, London SW17 0RE, UK; 2 Division of Infection and Immunity, Cardiff University School of Medicine, Heath Park, Cardiff CF14 4XN, UK; 3 Department of Medicine, St. Mary’s Hospital, Imperial College London, London W2 1PG, UK; 4 Division of Cancer and Genetics, Cardiff University School of Medicine, Heath Park, Cardiff CF14 4XN, UK; 5 Sorbonne Universités, UPMC Université Paris 06, Centre d’Immunologie et des Maladies Infectieuses (CIMI-Paris), 75013 Paris, France; 6 INSERM U1135, CIMI-Paris, 75013 Paris, France; 7 Vaccine Research Center, National Institute of Allergy and Infectious Diseases, National Institutes of Health, Bethesda, MD 20892, USA; 8 St. George’s University Hospitals National Health Service Foundation Trust, Blackshaw Road, London SW17 0QT, UK

**Keywords:** adaptive immunity, memory T cells, stem cell-like memory T cells, CD4^+^ T cells, CD8^+^ T cells, in vivo heavy water labeling, proliferation, telomere length, telomerase activity, memory T cell maintenance

## Abstract

Adaptive immunity requires the generation of memory T cells from naive precursors selected in the thymus. The key intermediaries in this process are stem cell-like memory T (T_SCM_) cells, multipotent progenitors that can both self-renew and replenish more differentiated subsets of memory T cells. In theory, antigen specificity within the T_SCM_ pool may be imprinted statically as a function of largely dormant cells and/or retained dynamically by more transitory subpopulations. To explore the origins of immunological memory, we measured the turnover of T_SCM_ cells in vivo using stable isotope labeling with heavy water. The data indicate that T_SCM_ cells in both young and elderly subjects are maintained by ongoing proliferation. In line with this finding, T_SCM_ cells displayed limited telomere length erosion coupled with high expression levels of active telomerase and Ki67. Collectively, these observations show that T_SCM_ cells exist in a state of perpetual flux throughout the human lifespan.

## Highlights

• Human stem cell-like memory T (T_SCM_) cells proliferate extensively in vivo
• Human T_SCM_ cells express high levels of Ki67
• Human T_SCM_ cells have long telomeres
• Human T_SCM_ cells display high levels of telomerase activity

## Graphical Abstract

**Figure:**
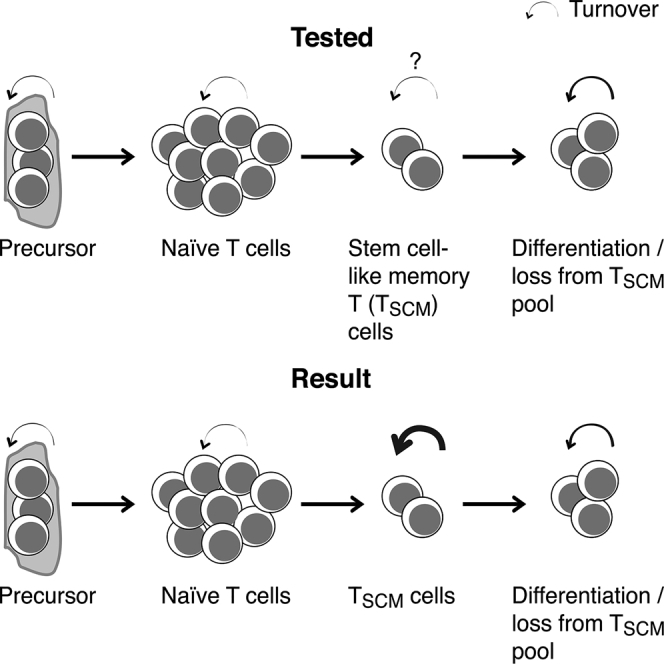

## Teaser

Stem cell-like memory T (T_SCM_) cells are multipotent progenitors that can both self-renew and replenish more differentiated subsets of memory T cells. Ahmed et al. find that human T_SCM_ cells are maintained by ongoing proliferation and display limited telomere length erosion coupled with high expression levels of active telomerase and Ki67.

## Introduction

Antigen encounter drives the formation of heterogeneous memory T cell populations, which deploy various effector functions with accelerated kinetics to ensure long-term protective immunity (Chang et al., 2014, Farber et al., 2014). The recently described stem cell-like memory T (T_SCM_) subset typically comprises 2%–3% of the circulating T cell pool and can be identified within a naive-like phenotype (CD45RA^+^CD45RO^–^CCR7^+^CD62L^+^CD27^+^CD28^+^) by expression of the memory marker CD95 (Gattinoni et al., 2011). In accordance with this definition, T_SCM_ cells mount anamnestic responses and display gene transcript profiles encompassing features of both naive T (T_N_) and central memory T (T_CM_) cells (Gattinoni et al., 2011). Moreover, T_SCM_ cells are endowed with considerable proliferative reserves and can differentiate in vitro and in vivo to reconstitute the entire spectrum of classically delineated memory T cells (Gattinoni et al., 2011). These characteristics suggest an antecedent role for T_SCM_ cells in the complex antigen-driven processes that ultimately capture and preserve immunological memories.

It is established that T_SCM_ cells persist at stable frequencies throughout the human lifespan (Di Benedetto et al., 2015). However, the mechanisms that underlie this remarkable longevity are incompletely defined. Two mutually non-exclusive possibilities exist: (1) T_SCM_ cells may endure under conditions of relative dormancy with prolonged survival; and/or (2) the T_SCM_ pool may be sustained by ongoing proliferation and cell turnover. In this study, we provide evidence consistent with the latter scenario and demonstrate that T_SCM_ cells are maintained in a state of dynamic flux.

## Results and Discussion

**Figure 1:**
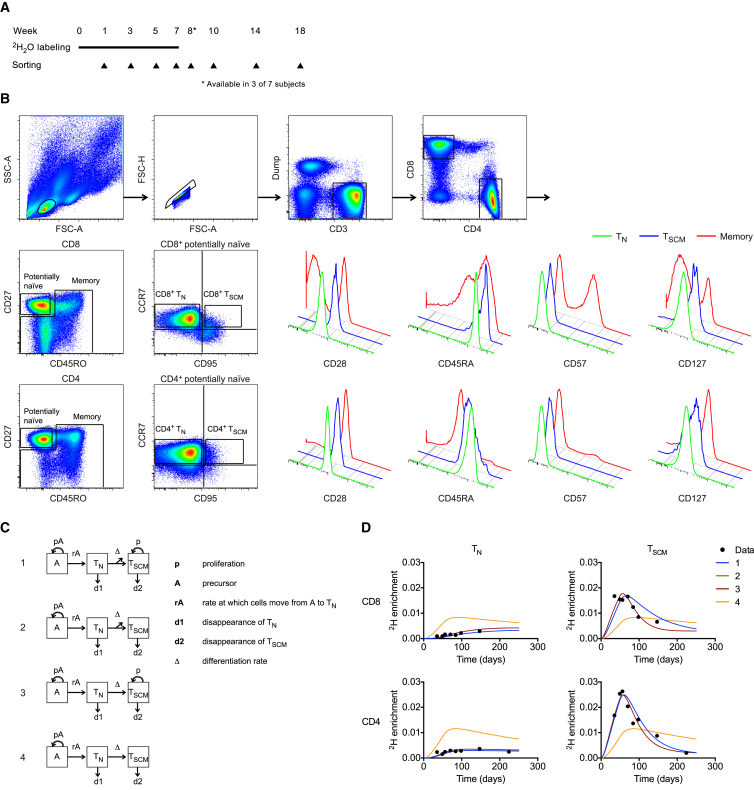
Label Incorporation in Naive and Stem Cell-like Memory T Cells (A) Schematic representation of the ^2^H_2_O-labeling protocol and sampling time points. (B) Successive panels depict the flow cytometric gating strategy used to sort CD4^+^ and CD8^+^ T_N_ and T_SCM_ cells. Lymphocytes were identified in a forward-scatter versus side-scatter plot, and single cells were resolved in a forward-scatter-height versus forward-scatter-area plot. Boolean gates were drawn for analysis only to exclude fluorochrome aggregates. Live CD3^+^CD14^–^CD19^–^ cells were assigned to the CD4^+^ or CD8^+^ lineage, and potentially naive CD27^bright^CD45RO^–^ cells were separated from memory T cells. Sort gates were then fixed on CCR7^+^CD95^–^ T_N_ cells and CCR7^+^CD95^+^ T_SCM_ cells. Histogram overlays show expression of CD28, CD45RA, CD57, and CD127 in the T_N_, T_SCM_, and memory subsets. (C) Schematic representation of the mathematical models applied to the labeling data. In the depicted variation, a precursor compartment replenishes T_N_ cells, which do not proliferate. Two further variations were considered, one eliminating the precursor compartment, and the other assuming T_N_ cell proliferation. Similar results were obtained with all three variations. (D) Experimental labeling data (black filled circles) and modeled curve fits for subject DW01 (young adult). The curve fits for model 1 overlie the curve fits for model 2.

**Figure 2:**
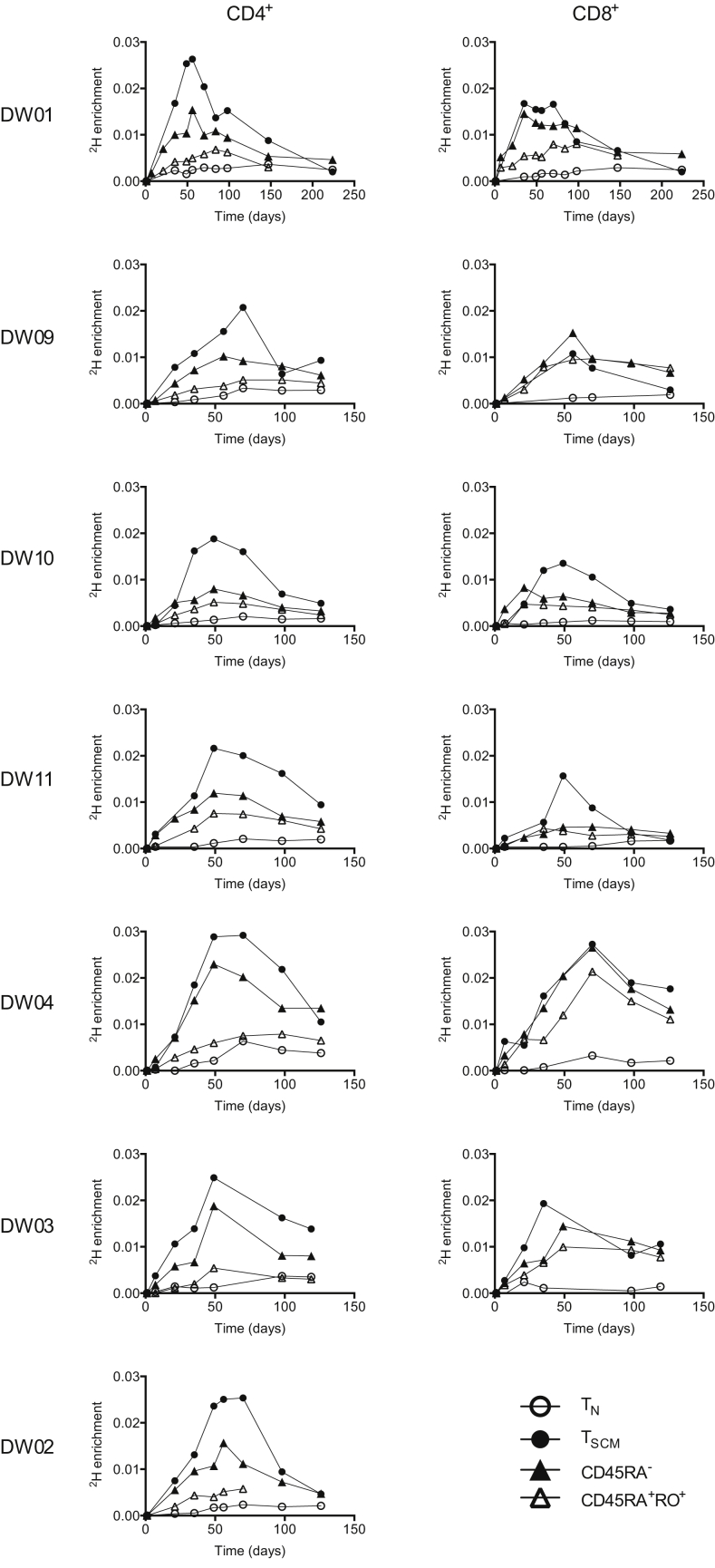
Comparative Label Enrichment in Naive, Stem Cell-like Memory and Other Memory T Cells Experimental labeling data for T_N_, T_SCM_, CD45RA^–^ memory, and CD45RA^+^CD45RO^+^ transitional memory T cells from subjects DW01, DW09, DW10, and DW11 (young adults), and DW04, DW03, and DW02 (elderly).

To investigate how T_SCM_ cells are maintained in humans, we conducted a long-term (7-week) stable isotope (^2^H_2_O) labeling study (Figure 1A). Deuterium (^2^H) enrichment of DNA extracted from rigorously sort-purified T cell subsets (Figure 1B) was measured at defined intervals using gas chromatography/mass spectrometry (Neese et al., 2002, Busch et al., 2007). CD4^+^ and CD8^+^ T_SCM_ cells rapidly incorporated ^2^H during the labeling phase and lost ^2^H during the delabeling phase (Figures 1D and S1). Moreover, the fractions of labeled CD4^+^ and CD8^+^ T_SCM_ cells were higher in the majority of subjects compared with the corresponding lineage-defined CD45RA^–^ and CD45RA^+^CD45RO^+^ memory T cells (Figure 2). Consistent with previous reports (Hellerstein et al., 2003, Ladell et al., 2008, Vrisekoop et al., 2008), we found only low levels of ^2^H enrichment in the T_N_ subset. These cells accumulated further label after ^2^H_2_O administration was discontinued, likely reflecting T_N_ cell proliferation in lymphoid tissue with delayed exit into the peripheral blood (Hellerstein et al., 2003). Given that ^2^H is incorporated into newly synthesized DNA generated during cell division, this dataset suggests that T_SCM_ cells are maintained in vivo by extensive proliferation.

To explore the source of label enrichment within the T_SCM_ pool, we considered four mathematical models of linear differentiation (Figure 1C). Two scenarios were postulated for T_SCM_ cells (dividing or non-dividing), and two scenarios were postulated for T_N_ cells (differentiation is accompanied or not accompanied by division, with the latter assuming that one T_N_ cell gives rise to one T_SCM_ cell). The model in which neither T_N_ nor T_SCM_ cells were free to proliferate could be excluded on the basis of the labeling data (Figures 1D and S1). Although it was not possible to separate the remaining models, all three indicated considerable replacement rates for the T_SCM_ population across lineages and subjects (median, 0.02 per day; inter-quartile range, 0.016–0.037 per day). These findings concur with the empirical view that recurrent cell division sustains the T_SCM_ compartment.

**Figure 3:**
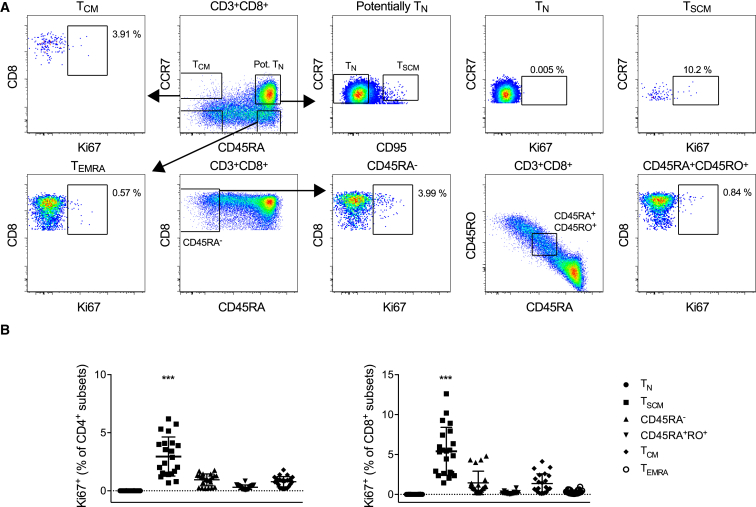
Ki67 Expression in Naive, Stem Cell-like Memory, and Other Memory T Cells (A) Intracellular Ki67 expression in the depicted T cell subsets from subject DW01 (young adult). Live CD3^+^CD14^–^CD19^–^ lymphocytes within the CD4^+^ and CD8^+^ lineages were identified as shown in Figure 1B. Conservative gates were placed around CCR7^+^CD95^–^ T_N_ cells and CCR7^+^CD95^+^ T_SCM_ cells within a naive-like phenotype (CD45RA^bright^CCR7^+^). (B) Intracellular Ki67 expression in CD4^+^ (left) and CD8^+^ (right) T cell subsets from healthy adult volunteers and subject DW01 (young adult). Peripheral blood mononuclear cells were stained in triplicate directly ex vivo. Horizontal bars represent mean values with SEs. T_CM_ (CD45RA^–^CCR7^+^); T_EMRA_ (CD45RA^+^CCR7^–^). Significance was assessed using a two-tailed Mann-Whitney test. Asterisks indicate p < 0.001 for all comparisons.

To substantiate this conclusion, we measured the expression of Ki67, which is limited to active phases of the cell cycle (Scholzen and Gerdes, 2000). High frequencies of Ki67^+^ T_SCM_ cells were detected in both the CD4^+^ and CD8^+^ lineages (Figures 3A and 3B). In contrast, Ki67^+^ events were rare in the corresponding T_N_ populations. A similar dichotomy prevails in macaques (Lugli et al., 2013). It has been shown previously that T_N_ cells can divide and retain a naive-like phenotype (Hellerstein et al., 2003, Ladell et al., 2008, Ladell et al., 2015). Proliferation is therefore not necessarily linked with differentiation, a finding that also holds for T_SCM_ cells in vitro under certain conditions (Gattinoni et al., 2011). Moreover, T_SCM_ cells stimulated with the homeostatic cytokine interleukin (IL)-15 in vitro can divide repeatedly over 10 days, whereas T_N_ cells generally divide once or twice up to a maximum of four times in the same period (Gattinoni et al., 2011). These considerations support a model of self-renewal within the T_SCM_ pool.

**Figure 4:**
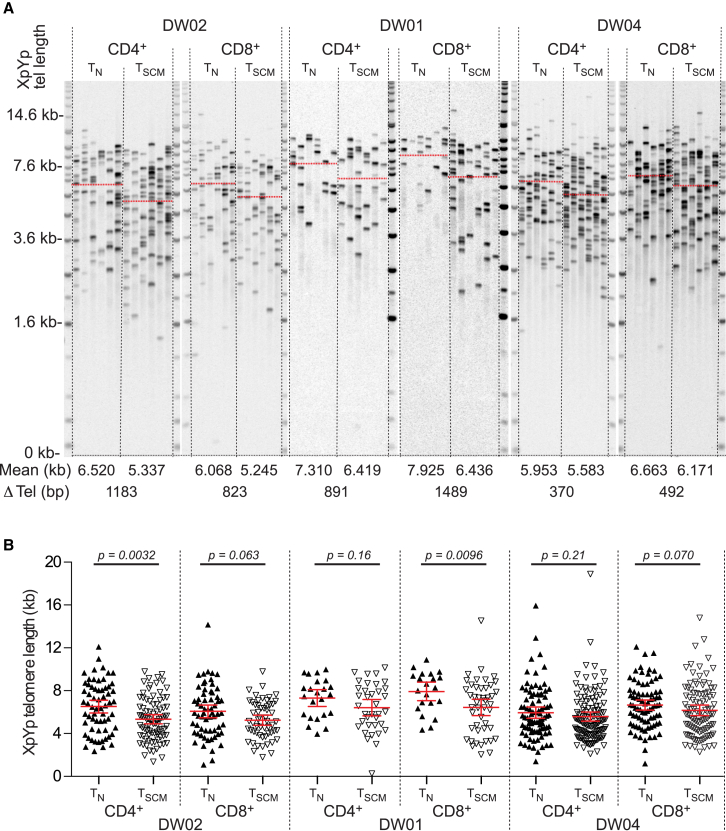
Telomere Lengths in Naive and Stem Cell-like Memory T Cells (A) Representative single telomere length analysis data from subjects DW02 (elderly), DW01 (young adult), and DW04 (elderly). Single telomere length analysis was conducted at the XpYp telomere for CD4^+^ and CD8^+^ T_N_ and T_SCM_ cells. Mean values and telomere length differentials are shown (bottom). (B) XpYp telomere length distributions as scatterplots. Significance was assessed using a two-tailed Mann-Whitney test.

To corroborate the finding that T_SCM_ cells manifest higher rates of turnover in vivo relative to T_N_ cells, we used single telomere length analysis (Baird et al., 2003) to determine the replicative history of these distinct subsets (Figures 4A and S2A). Individual telomere lengths were distributed around a significantly lower mean in the T_SCM_ population compared with the T_N_ population (CD4^+^ T cells, p = 0.0002; CD8^+^ T cells, p = 0.0007; two-tailed Mann-Whitney p values pooled by Fisher’s method) (Figures 4B and S2B). Moreover, T_SCM_ cells displayed higher levels of telomerase activity than either T_N_ or other memory T cells (Figure S2C). In the absence of telomerase activity, telomeres erode by 90 bp each time a population doubles in size (Baird et al., 2003). The telomere length differentials between T_SCM_ and T_N_ cells ranged from 370 to 1,489 bp (mean, 787 bp; Figures 4A, 4B, S2A, and S2B), equivalent to a maximum of almost 17 doublings at the population level. However, the true proliferative disparity will be substantially larger because telomerase markedly slows the rate of telomere erosion. These data are again indicative of considerable turnover within the T_SCM_ compartment and further suggest a biological requirement for self-maintenance.

It remains unclear whether the T cell differentiation pathway is linear or bifurcated, with the latter model proposing that a single T_N_ cell gives rise to both a short-lived effector and a long-lived memory T cell (Arsenio et al., 2015, Flossdorf et al., 2015). There is some evidence for asymmetric division within the T_N_ pool (Chang et al., 2007, Arsenio et al., 2014), whereas other reports ascribe stemness to the T_CM_ pool (Graef et al., 2014). Irrespective of this ongoing debate, T_SCM_ cells are ideally equipped to amplify and preserve clonotypically encoded immunological memories (Gattinoni et al., 2011). In simian immunodeficiency virus-infected macaques, antigen-specific T_SCM_ cells display a 10-fold greater capacity to survive compared with T_CM_ cells following the loss of cognate antigen (Lugli et al., 2013). Similarly, vaccine-induced T_SCM_ cells can persist for decades with a naive-like profile (Fuertes Marraco et al., 2015). The T_SCM_ compartment is also preserved in HIV-infected individuals on long-term anti-retroviral therapy (Vigano et al., 2015), despite the presence of a latent viral reservoir in the CD4^+^ lineage (Jaafoura et al., 2014, Buzon et al., 2014). Further evidence attests to the proliferative capacity of T_SCM_ cells. In humans, the administration of cyclophosphamide after allogeneic bone marrow transplantation eradicates T_SCM_ cells, but leaves the T_N_ compartment largely intact (Roberto et al., 2015). Moreover, immune reconstitution is preferentially driven by T_SCM_ cells, at least in mice (Gattinoni et al., 2011). It therefore seems likely that the rapid turnover of T_SCM_ cells at the whole-population level reflects a composite of kinetically distinct subsets, potentially dissociated by transcriptional integration of variable antigenic stimuli and other immune activation signals (Cartwright et al., 2014, Lugli et al., 2013, Roychoudhuri et al., 2016). The data presented here are consistent with such divergent outcomes and suggest that nascent immunological memory is encapsulated within fluid cellular networks.

## Experimental Procedures

### Human Samples

Seven healthy adults participated in the labeling study. Recruitment was stratified to include both young (aged 29–47 years) and elderly (aged 64–83 years) subjects, all of whom tested seropositive for cytomegalovirus and seronegative for HIV. Further peripheral blood samples were obtained from healthy adult volunteers. Approval was granted by the Cardiff University School of Medicine and London-Chelsea Research Ethics Committees. All studies were conducted according to the principles of the Declaration of Helsinki.

### In Vivo Labeling

Study participants ingested small doses of 70% deuterated water (^2^H_2_O) over a 7-week period (50 ml three times daily for 1 week, then twice daily thereafter). Saliva samples were collected weekly for evaluation of body water labeling rates. Peripheral blood was collected at baseline and then at weeks 1, 3, 5, 7, 8, 10, 14, and 18. In one case (DW01), two further samples were collected (weeks 21 and 32).

### Flow Cytometry and Cell Sorting

Peripheral blood mononuclear cells were isolated using standard density gradient centrifugation and stained with Live/Dead Fixable Aqua (Life Technologies), and anti-CD14-V500 and anti-CD19-V500 (BD Horizon) to exclude irrelevant signals from the analysis. The following monoclonal antibodies (mAbs) were used in further stains: (1) anti-CD3-H7APC, anti-CD28-APC, anti-CD45RA-PE, and anti-CD57-FITC (BD Pharmingen); (2) anti-CD4-Cy5.5PE and anti-CD27-QD605 (Life Technologies); (3) anti-CD45RO-ECD (Beckman Coulter); and (4) anti-CD8-BV711, anti-CD127-BV421, and anti-PD-1-BV421 (BioLegend). Naive (CD27^bright^CD45RO^–^CCR7^+^CD95^–^), stem cell-like memory (CD27^bright^CD45RO^–^CCR7^+^CD95^+^), transitional memory (CD45RA^+^CD45RO^+^), and memory (CD45RA^–^) CD4^+^ and CD8^+^ T cells were sorted at >98% purity using a custom-modified FACSAria II flow cytometer (BD Biosciences). Intracellular expression of Ki67 was evaluated separately using an Alexa Fluor 647-conjugated mAb in conjunction with a Cytofix/Cytoperm Kit (BD Biosciences). Data were analyzed with FlowJo software, version 9.7.6 (Tree Star).

### Measurement and Analysis of ^2^H Enrichment in T Cell DNA

The stable isotope-based method for measuring T cell proliferation has been described previously (Hellerstein et al., 1999, McCune et al., 2000, Neese et al., 2001). Additional precautions and controls were incorporated to ensure the accurate quantification of ^2^H enrichment in low-abundance samples (Busch et al., 2007). Briefly, DNA from sort-purified T cell subsets was released by boiling and hydrolyzed according to standard protocols. Deoxyribonucleosides were derivatized using pentafluorobenzyl hydroxylamine (Sigma-Aldrich). Gas chromatography/mass spectrometry (Agilent 5873/6980) was performed in negative chemical ionization mode using a DB-17 column (J&W Scientific; Agilent). The M+1/M+0 isotopomer ratio was monitored at mass-to-charge (*m/z*) 436/435. To normalize for body water enrichment, weekly saliva samples were analyzed for ^2^H_2_O content via calcium carbide-induced acetylene generation, monitoring at *m/z* 27/26 (Previs et al., 1996).

### Single Telomere Length Analysis

DNA was extracted from 3,000 sort-purified T cells using a QIAmp DNA Micro Kit (QIAGEN). Single telomere length analysis was carried out at the XpYp telomere as described previously (Capper et al., 2007). Briefly, 1 μM of the Telorette-2 linker was added to purified genomic DNA in a final volume of 40 μL per sample. Multiple PCRs were performed for each test DNA in 10-μL volumes incorporating 250 pg of DNA and 0.5 μM of the telomere-adjacent and Teltail primers in 75 mM Tris-HCl pH 8.8, 20 mM (NH_4_)_2_SO_4_, 0.01% Tween-20, and 1.5 mM MgCl_2_, with 0.5 U of a 10:1 mixture of Taq (ABGene) and Pwo polymerase (Roche Molecular Biochemicals). The reactions were processed in a Tetrad2 Thermal Cycler (Bio-Rad). DNA fragments were resolved by 0.5% Tris-acetate-EDTA agarose gel electrophoresis and identified by Southern hybridization with a random-primed α-^33^P-labeled (PerkinElmer) TTAGGG repeat probe, together with probes specific for the 1-kb (Stratagene) and 2.5-kb (Bio-Rad) molecular weight markers. Hybridized fragments were detected using a Typhoon FLA 9500 Phosphorimager (GE Healthcare). The molecular weights of the DNA fragments were calculated using a Phoretix 1D Quantifier (Nonlinear Dynamics).

### Telomerase Activity

Sort-purified T cells were lyzed and assayed in two steps using a modified SYBR Green real-time quantitative telomerase repeat amplification protocol (Wege et al., 2003). Standard curves were obtained from serial dilutions of a 293T cell extract with known telomerase activity. Experimental telomerase activity was calculated with reference to 293T cells and expressed as relative telomerase activity (Ct_293T_/Ct_sample_).

### Mathematical Modeling

Four mathematical models describing the relationship between T_N_ and T_SCM_ cells were constructed using ordinary differential equations and fitted to the labeling data in R. Two variations were also considered for each model: (1) proliferation was factored into the peripheral blood T_N_ pool; and (2) the precursor compartment was omitted for T_N_ cells. None of these variants yielded better predictions than the original models. The following equations were used to describe the rate of change of the fraction of labeled DNA in the precursor and T_N_ compartments:

$${\dot{F}}_{A}=r_{A}\left(cU-F_{A}\right)$$

$${\dot{F}}_{TN}=\left(d_{1}+\Delta \right)\left(F_{A}-F_{TN}\right),$$

where $r_{A}$ represents the rate at which naive cells move from *A* to $T_{N}$, $d_{1}$ is the disappearance rate of T_N_ cells in the blood, Δ is the differentiation rate (associated with proliferation in models 1 and 2) of T_N_ into T_SCM_ cells, *c* is the amplification factor for enrichment, and *U* is the function describing labeling and delabeling in saliva. The following equations were used for the T_SCM_ pool:

$$\text{Model}\;1:{\dot{F}}_{TSCM}=\frac{\Delta T_{N}}{T_{SCM}}\left(cU+F_{N}\right)+pcU-\left(\frac{2\Delta T_{N}}{T_{SCM}}+p\right)F_{TSCM}$$

$$\text{Model}\;2:{\dot{F}}_{TSCM}=\frac{\Delta T_{N}}{T_{SCM}}\left(cU+F_{N}-2F_{TSCM}\right)$$

$$\text{Model}\;3:{\dot{F}}_{TSCM}=\frac{\Delta T_{N}}{T_{SCM}}\;F_{N}+pcU-\left(\frac{\Delta T_{N}}{T_{SCM}}+p\right)F_{TSCM}$$

$$\text{Model}\;4:{\dot{F}}_{TSCM}=\frac{\Delta T_{N}}{T_{SCM}}\left(F_{N}-F_{TSCM}\right),$$

where p is the rate of proliferation within the T_SCM_ pool and $T_{N}/T_{SCM}$ is the ratio of the sizes of the T_N_ and T_SCM_ pools measured experimentally. Model fits were compared using the corrected Akaike information criterion (Burnham and Anderson, 2002).

### Statistical Analysis

Telomere lengths between the T_N_ and T_SCM_ populations were compared using a two-tailed Mann-Whitney test. The p values were pooled using Fisher’s method.

## Author Contributions

R.A., L.R., K.L.M., R.E.J., T.F., Y.Z., and K.L. carried out experiments. R.A., L.R., R.E.J., V.A., D.M.B., D.C.M., and K.L. analyzed data. P.C.d.A., L.B., M.E., and B.A. modeled data. D.A.P., D.C.M., and K.L. designed experiments. P.C.d.A., L.B., V.A., D.M.B., and B.A. edited the manuscript. D.A.P., D.C.M., and K.L. wrote the manuscript.

## Supplemental Information

Document S1. Figures S1 and S2

Document S2. Article plus Supplemental Information
