# Supplementary material for: Human Stem Cell-like Memory T Cells Are Maintained in a State of Dynamic Flux
Source: Cell Rep. 2016 Dec 13;17(11):2811–8. doi: 10.1016/j.celrep.2016.11.037 (PMC5186732; doi:10.1016/j.celrep.2016.11.037)
Supplement: Document S1. Figures S1 and S2 [file mmc1.pdf]

Cell Reports, Volume *17*

Supplemental Information

Human Stem Cell-like Memory T Cells Are Maintained in a State of Dynamic Flux

Raya Ahmed, Laureline Roger, Pedro Costa del Amo, Kelly L. Miners, Rhiannon E. Jones, Lies Boelen, Tinhinane Fali, Marjet Elemans, Yan Zhang, Victor Appay, Duncan M. Baird, Becca Asquith, David A. Price, Derek C. Macallan, and Kristin Ladell

Supplemental Figure 1 (related to Figure 1):

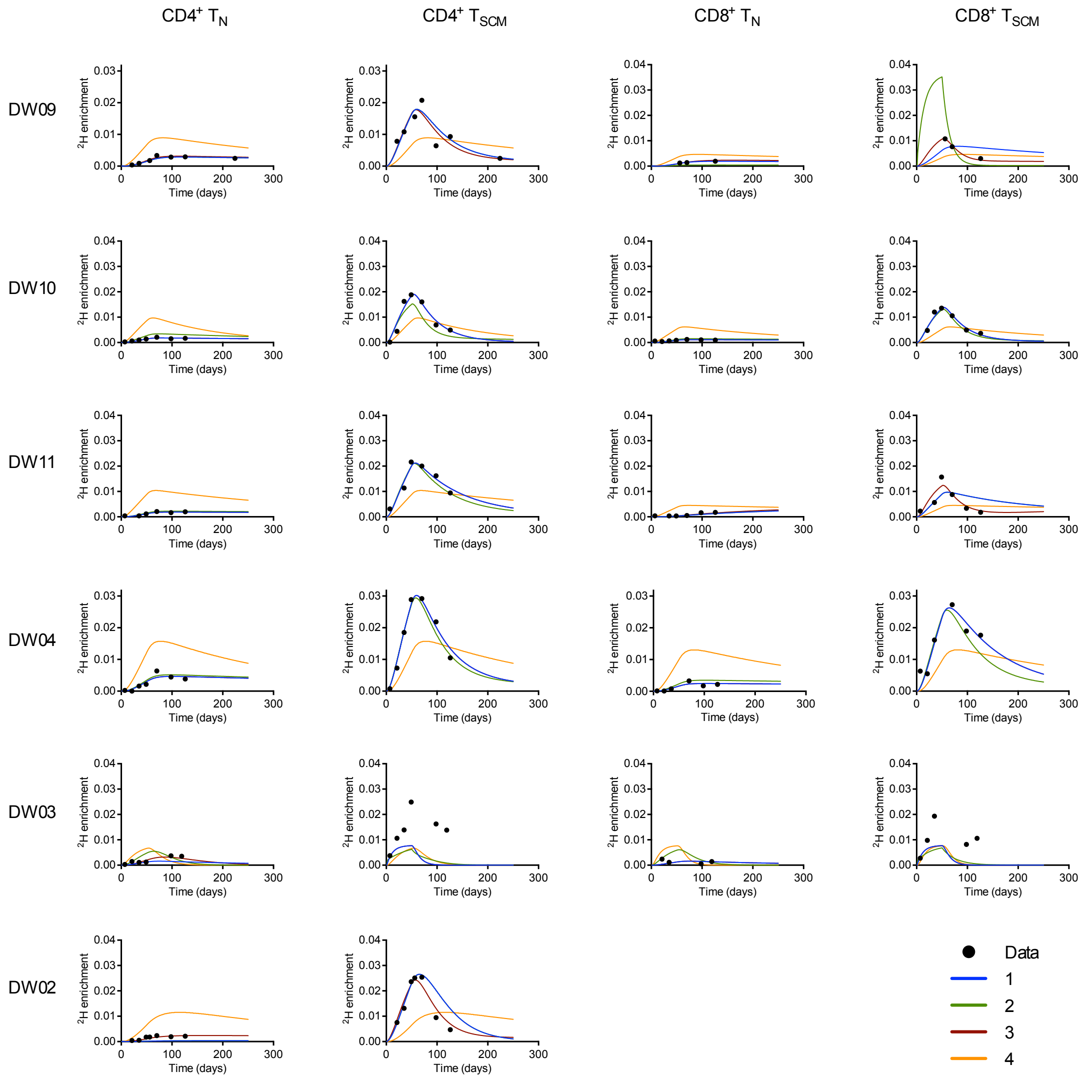

Supplemental Figure 1. Label incorporation in naïve and stem cell-like memory T cells. Experimental labeling data (black filled circles) and modeled curve fits for subjects DW09, DW10 and DW11 (young adults), and DW04, DW03 and DW02 (elderly).

Supplemental Figure 2 (related to Figure 4):

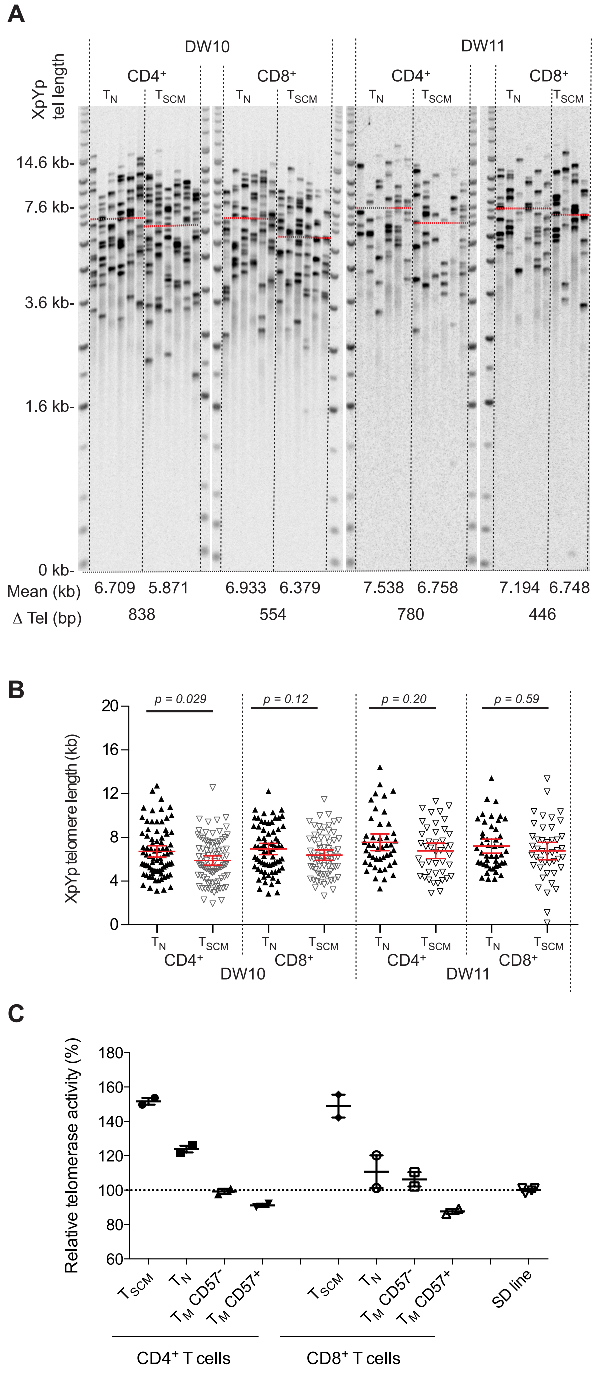

Supplemental Figure 2. Telomere lengths and telomerase activity in naïve and stem cell-like memory T cells. (A) Representative STELA data from subjects DW10 and DW11 (young adults). STELA was conducted at the XpYp telomere for $CD4^{+}$ and $CD8^{+}$ T_N and T_{SCM} cells. Mean values and telomere length differentials are shown (bottom). (B) XpYp telomere length distributions as scatter plots. Significance was

assessed using a two-tailed Mann-Whitney test. (C) Relative telomerase activity for $CD4^+$ and $CD8^+$ T_N, T_{SCM} and memory T (T_M) cells from subjects DW02 and DW04 (elderly). T_M cells are segregated as $CD57^-$ (less differentiated) and $CD57^+$ (more differentiated). Horizontal bars represent mean values with standard errors.
