# Supplementary material for: Human Stem Cell-like Memory T Cells Are Maintained in a State of Dynamic Flux
Source: Cell Rep. 2016 Dec 13;17(11):2811–8. doi: 10.1016/j.celrep.2016.11.037 (PMC5186732; doi:10.1016/j.celrep.2016.11.037)
Supplement: Document S2. Article plus Supplemental Information [file mmc2.pdf]

Cell Reports

Report

Human Stem Cell-like Memory T Cells Are Maintained in a State of Dynamic Flux

Graphical Abstract

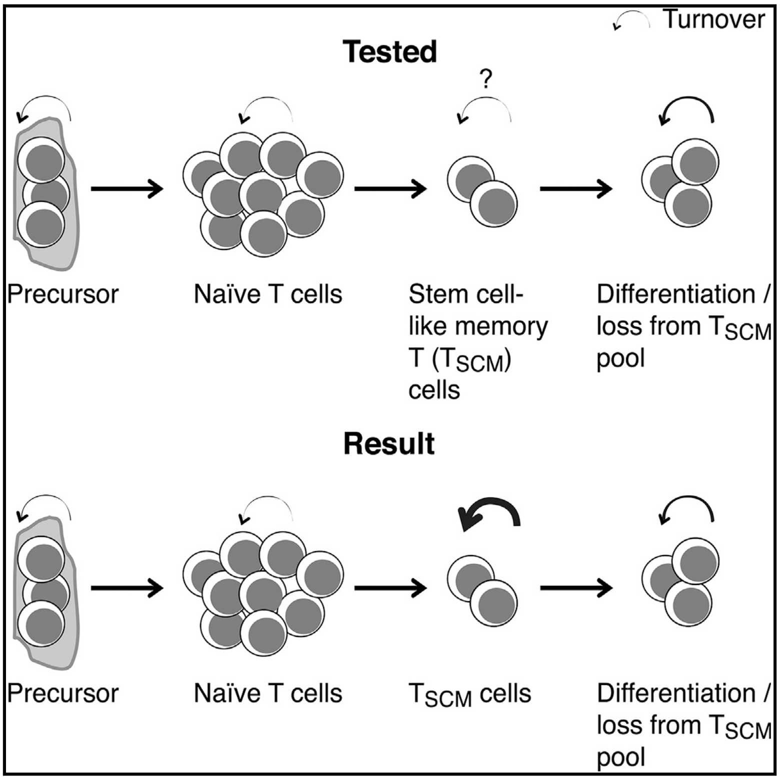

Authors

Raya Ahmed, Laureline Roger, Pedro Costa del Amo, ..., David A. Price, Derek C. Macallan, Kristin Ladell

Correspondence

priced6@cardiff.ac.uk (D.A.P.),
macallan@sgul.ac.uk (D.C.M.),
ladellk@gmail.com (K.L.)

In Brief

Stem cell-like memory T (T_{SCM}) cells are multipotent progenitors that can both self-renew and replenish more differentiated subsets of memory T cells. Ahmed et al. find that human T_{SCM} cells are maintained by ongoing proliferation and display limited telomere length erosion coupled with high expression levels of active telomerase and Ki67.

Highlights

- Human stem cell-like memory T (T_{SCM}) cells proliferate extensively in vivo
- Human T_{SCM} cells express high levels of Ki67
- Human T_{SCM} cells have long telomeres
- Human T_{SCM} cells display high levels of telomerase activity

Ahmed et al., 2016, Cell Reports *17*, 2811–2818
December 13, 2016 © 2016 The Authors.
http://dx.doi.org/10.1016/j.celrep.2016.11.037

CellPress

Cell Reports
Report

OPEN ACCESS
CellPress

Human Stem Cell-like Memory T Cells Are Maintained in a State of Dynamic Flux

Raya Ahmed,[1] Laureline Roger,[2] Pedro Costa del Amo,[3] Kelly L. Miners,[2] Rhiannon E. Jones,[4] Lies Boelen,[3] Tinhinane Fali,[5,6] Marjet Elemans,[3] Yan Zhang,[1] Victor Appay,[5,6] Duncan M. Baird,[4] Becca Asquith,[3] David A. Price,[2,7,*] Derek C. Macallan,[1,8,*] and Kristin Ladell[2,9,*]
[1]Institute for Infection and Immunity, St. George's, University of London, London SW17 0RE, UK
[2]Division of Infection and Immunity, Cardiff University School of Medicine, Heath Park, Cardiff CF14 4XN, UK
[3]Department of Medicine, St. Mary's Hospital, Imperial College London, London W2 1PG, UK
[4]Division of Cancer and Genetics, Cardiff University School of Medicine, Heath Park, Cardiff CF14 4XN, UK
[5]Sorbonne Universités, UPMC Université Paris 06, Centre d'Immunologie et des Maladies Infectieuses (CIMI-Paris), 75013 Paris, France
[6]INSERM U1135, CIMI-Paris, 75013 Paris, France
[7]Vaccine Research Center, National Institute of Allergy and Infectious Diseases, National Institutes of Health, Bethesda, MD 20892, USA
[8]St. George's University Hospitals National Health Service Foundation Trust, Blackshaw Road, London SW17 0QT, UK
[9]Lead Contact
*Correspondence: priced6@cardiff.ac.uk (D.A.P.), macallan@sgul.ac.uk (D.C.M.), ladellk@gmail.com (K.L.)
http://dx.doi.org/10.1016/j.celrep.2016.11.037

SUMMARY

Adaptive immunity requires the generation of memory T cells from naive precursors selected in the thymus. The key intermediaries in this process are stem cell-like memory T (T_{SCM}) cells, multipotent progenitors that can both self-renew and replenish more differentiated subsets of memory T cells. In theory, antigen specificity within the T_{SCM} pool may be imprinted statically as a function of largely dormant cells and/or retained dynamically by more transitory subpopulations. To explore the origins of immunological memory, we measured the turnover of T_{SCM} cells in vivo using stable isotope labeling with heavy water. The data indicate that T_{SCM} cells in both young and elderly subjects are maintained by ongoing proliferation. In line with this finding, T_{SCM} cells displayed limited telomere length erosion coupled with high expression levels of active telomerase and Ki67. Collectively, these observations show that T_{SCM} cells exist in a state of perpetual flux throughout the human lifespan.

INTRODUCTION

Antigen encounter drives the formation of heterogeneous memory T cell populations, which deploy various effector functions with accelerated kinetics to ensure long-term protective immunity (Chang et al., 2014; Farber et al., 2014). The recently described stem cell-like memory T (T_{SCM}) subset typically comprises 2%–3% of the circulating T cell pool and can be identified within a naive-like phenotype ($CD45RA^+CD45RO^-CCR7^+$ $CD62L^+CD27^+CD28^+$) by expression of the memory marker CD95 (Gattinoni et al., 2011). In accordance with this definition, T_{SCM} cells mount anamnestic responses and display gene transcript profiles encompassing features of both naive T (T_N) and central memory T (T_{CM}) cells (Gattinoni et al., 2011). Moreover, T_{SCM} cells are endowed with considerable proliferative reserves and can differentiate in vitro and in vivo to reconstitute the entire spectrum of classically delineated memory T cells (Gattinoni et al., 2011). These characteristics suggest an antecedent role for T_{SCM} cells in the complex antigen-driven processes that ultimately capture and preserve immunological memories.

It is established that T_{SCM} cells persist at stable frequencies throughout the human lifespan (Di Benedetto et al., 2015). However, the mechanisms that underlie this remarkable longevity are incompletely defined. Two mutually non-exclusive possibilities exist: (1) T_{SCM} cells may endure under conditions of relative dormancy with prolonged survival; and/or (2) the T_{SCM} pool may be sustained by ongoing proliferation and cell turnover. In this study, we provide evidence consistent with the latter scenario and demonstrate that T_{SCM} cells are maintained in a state of dynamic flux.

RESULTS AND DISCUSSION

To investigate how T_{SCM} cells are maintained in humans, we conducted a long-term (7-week) stable isotope (^2H_2O) labeling study (Figure 1A). Deuterium (^2H) enrichment of DNA extracted from rigorously sort-purified T cell subsets (Figure 1B) was measured at defined intervals using gas chromatography/mass spectrometry (Neese et al., 2002; Busch et al., 2007). $CD4^+$ and $CD8^+$ T_{SCM} cells rapidly incorporated ^2H during the labeling phase and lost ^2H during the delabeling phase (Figures 1D and S1). Moreover, the fractions of labeled $CD4^+$ and $CD8^+$ T_{SCM} cells were higher in the majority of subjects compared with the corresponding lineage-defined $CD45RA^-$ and $CD45RA^+CD45RO^+$ memory T cells (Figure 2). Consistent with previous reports (Hellerstein et al., 2003; Ladell et al., 2008; Vrisekoop et al., 2008), we found only low levels of ^2H enrichment in the T_N subset. These cells accumulated further label after ^2H_2O administration was discontinued, likely reflecting T_N cell proliferation in lymphoid tissue with delayed exit into the peripheral blood (Hellerstein et al., 2003). Given that ^2H is incorporated into newly synthesized DNA

CrossMark

This is an open access article under the CC BY license (http://creativecommons.org/licenses/by/4.0/).

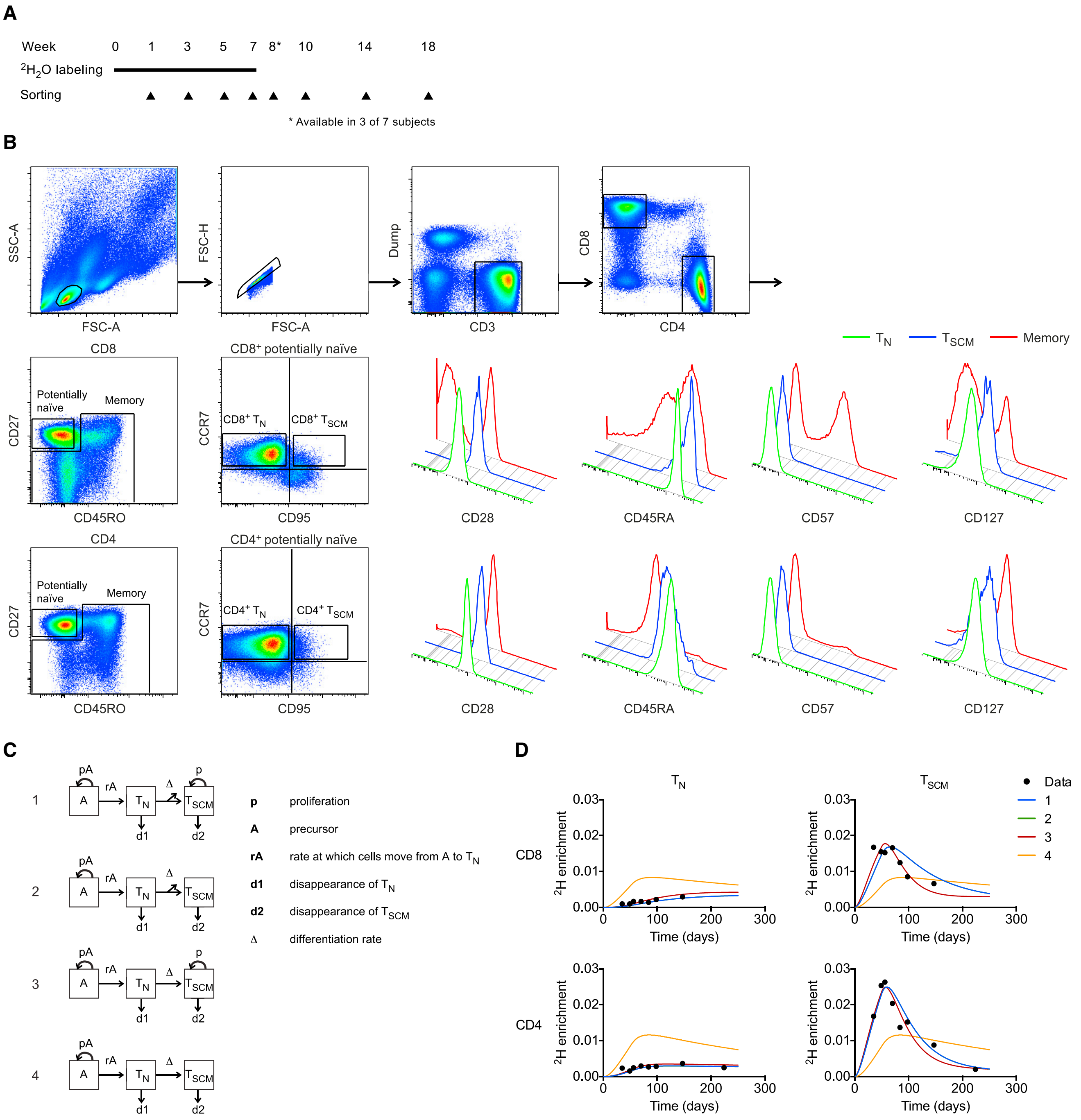

Figure 1. Label Incorporation in Naive and Stem Cell-like Memory T Cells

(A) Schematic representation of the $^{2}H_2O$-labeling protocol and sampling time points.

(B) Successive panels depict the flow cytometric gating strategy used to sort $CD4^{+}$ and $CD8^{+}$ T_N and T_{SCM} cells. Lymphocytes were identified in a forward-scatter versus side-scatter plot, and single cells were resolved in a forward-scatter-height versus forward-scatter-area plot. Boolean gates were drawn for analysis only to exclude fluorochrome aggregates. Live $CD3^{+}CD14^{-}CD19^{-}$ cells were assigned to the $CD4^{+}$ or $CD8^{+}$ lineage, and potentially naive $CD27^{bright}CD45RO^{-}$ cells were separated from memory T cells. Sort gates were then fixed on $CCR7^{+}CD95^{-}$ T_N cells and $CCR7^{+}CD95^{+}$ T_{SCM} cells. Histogram overlays show expression of CD28, CD45RA, CD57, and CD127 in the T_N, T_{SCM}, and memory subsets.

(C) Schematic representation of the mathematical models applied to the labeling data. In the depicted variation, a precursor compartment replenishes T_N cells, which do not proliferate. Two further variations were considered, one eliminating the precursor compartment, and the other assuming T_N cell proliferation. Similar results were obtained with all three variations.

(D) Experimental labeling data (black filled circles) and modeled curve fits for subject DW01 (young adult). The curve fits for model 1 overlie the curve fits for model 2.

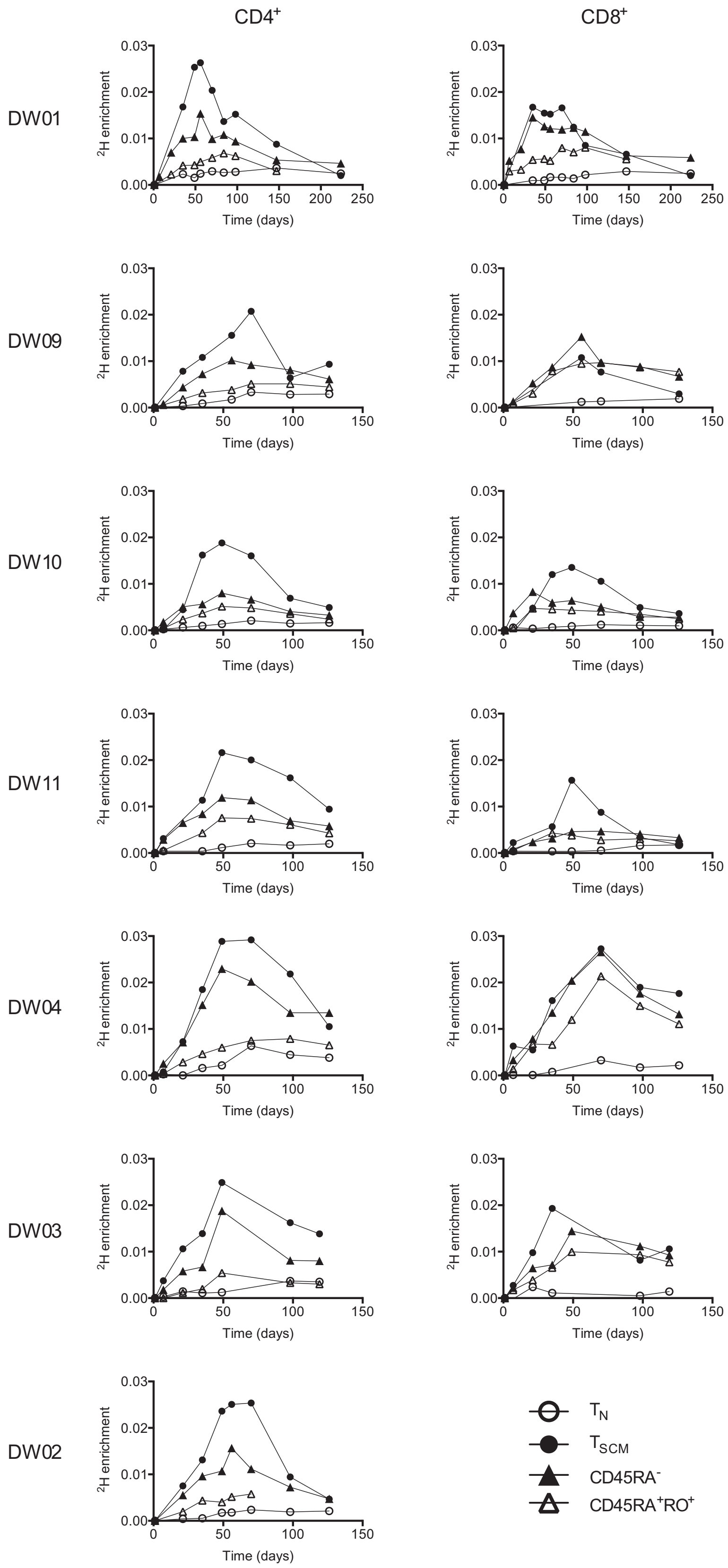

Figure 2. Comparative Label Enrichment in Naive, Stem Cell-like Memory and Other Memory T Cells

Experimental labeling data for T_N, T_{SCM}, CD45RA^{-} memory, and CD45RA^{+}CD45RO^{+} transitional memory T cells from subjects DW01, DW09, DW10, and DW11 (young adults), and DW04, DW03, and DW02 (elderly).

OPEN ACCESS
CellPress

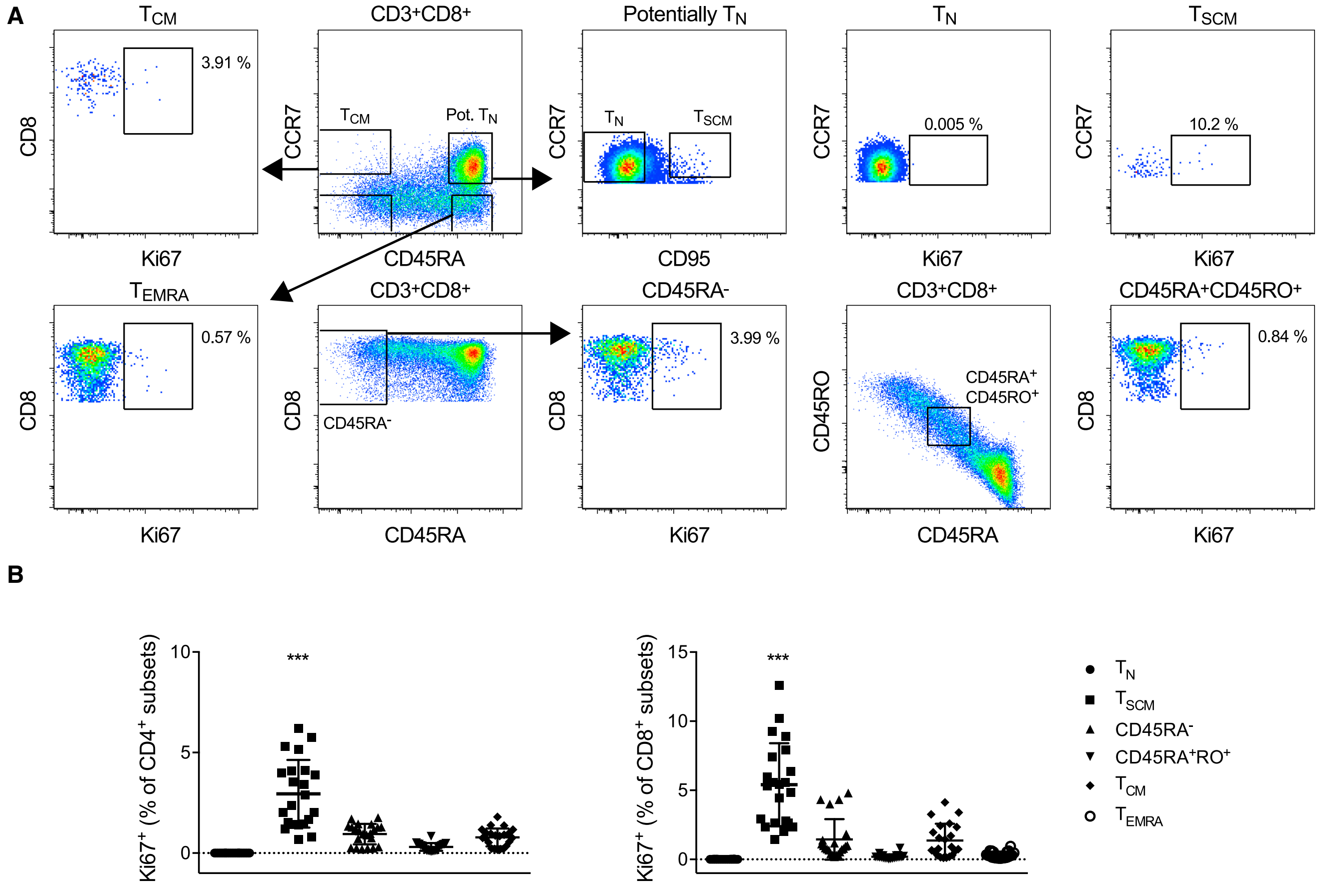

Figure 3. Ki67 Expression in Naive, Stem Cell-like Memory, and Other Memory T Cells

(A) Intracellular Ki67 expression in the depicted T cell subsets from subject DW01 (young adult). Live CD3$^+$CD14$^-$CD19$^-$ lymphocytes within the CD4$^+$ and CD8$^+$ lineages were identified as shown in Figure 1B. Conservative gates were placed around CCR7$^+$CD95$^-$ T$_N$ cells and CCR7$^+$CD95$^+$ T$_{SCM}$ cells within a naive-like phenotype (CD45RA^{bright}CCR7$^+$).

(B) Intracellular Ki67 expression in CD4$^+$ (left) and CD8$^+$ (right) T cell subsets from healthy adult volunteers and subject DW01 (young adult). Peripheral blood mononuclear cells were stained in triplicate directly ex vivo. Horizontal bars represent mean values with SEs. T$_{CM}$ (CD45RA$^-$CCR7$^+$); T$_{EMRA}$ (CD45RA$^+$CCR7$^-$). Significance was assessed using a two-tailed Mann-Whitney test. Asterisks indicate $p < 0.001$ for all comparisons.

generated during cell division, this dataset suggests that T$_{SCM}$ cells are maintained in vivo by extensive proliferation.

To explore the source of label enrichment within the T$_{SCM}$ pool, we considered four mathematical models of linear differentiation (Figure 1C). Two scenarios were postulated for T$_{SCM}$ cells (dividing or non-dividing), and two scenarios were postulated for T$_N$ cells (differentiation is accompanied or not accompanied by division, with the latter assuming that one T$_N$ cell gives rise to one T$_{SCM}$ cell). The model in which neither T$_N$ nor T$_{SCM}$ cells were free to proliferate could be excluded on the basis of the labeling data (Figures 1D and S1). Although it was not possible to separate the remaining models, all three indicated considerable replacement rates for the T$_{SCM}$ population across lineages and subjects (median, 0.02 per day; inter-quartile range, 0.016–0.037 per day). These findings concur with the empirical view that recurrent cell division sustains the T$_{SCM}$ compartment.

To substantiate this conclusion, we measured the expression of Ki67, which is limited to active phases of the cell cycle (Scholzen and Gerdes, 2000). High frequencies of Ki67$^+$ T$_{SCM}$ cells were detected in both the CD4$^+$ and CD8$^+$ lineages (Figures 3A and 3B). In contrast, Ki67$^+$ events were rare in the corresponding T$_N$ populations. A similar dichotomy prevails in macaques (Lugli et al., 2013). It has been shown previously that T$_N$ cells can divide and retain a naive-like phenotype (Hellerstein et al., 2003; Ladell et al., 2008, 2015). Proliferation is therefore not necessarily linked with differentiation, a finding that also holds for T$_{SCM}$ cells in vitro under certain conditions (Gattinoni et al., 2011). Moreover, T$_{SCM}$ cells stimulated with the homeostatic cytokine interleukin (IL)-15 in vitro can divide repeatedly over 10 days, whereas T$_N$ cells generally divide once or twice up to a maximum of four times in the same period (Gattinoni et al., 2011). These considerations support a model of self-renewal within the T$_{SCM}$ pool.

To corroborate the finding that T$_{SCM}$ cells manifest higher rates of turnover in vivo relative to T$_N$ cells, we used single telomere length analysis (Baird et al., 2003) to determine the replicative history of these distinct subsets (Figures 4A and

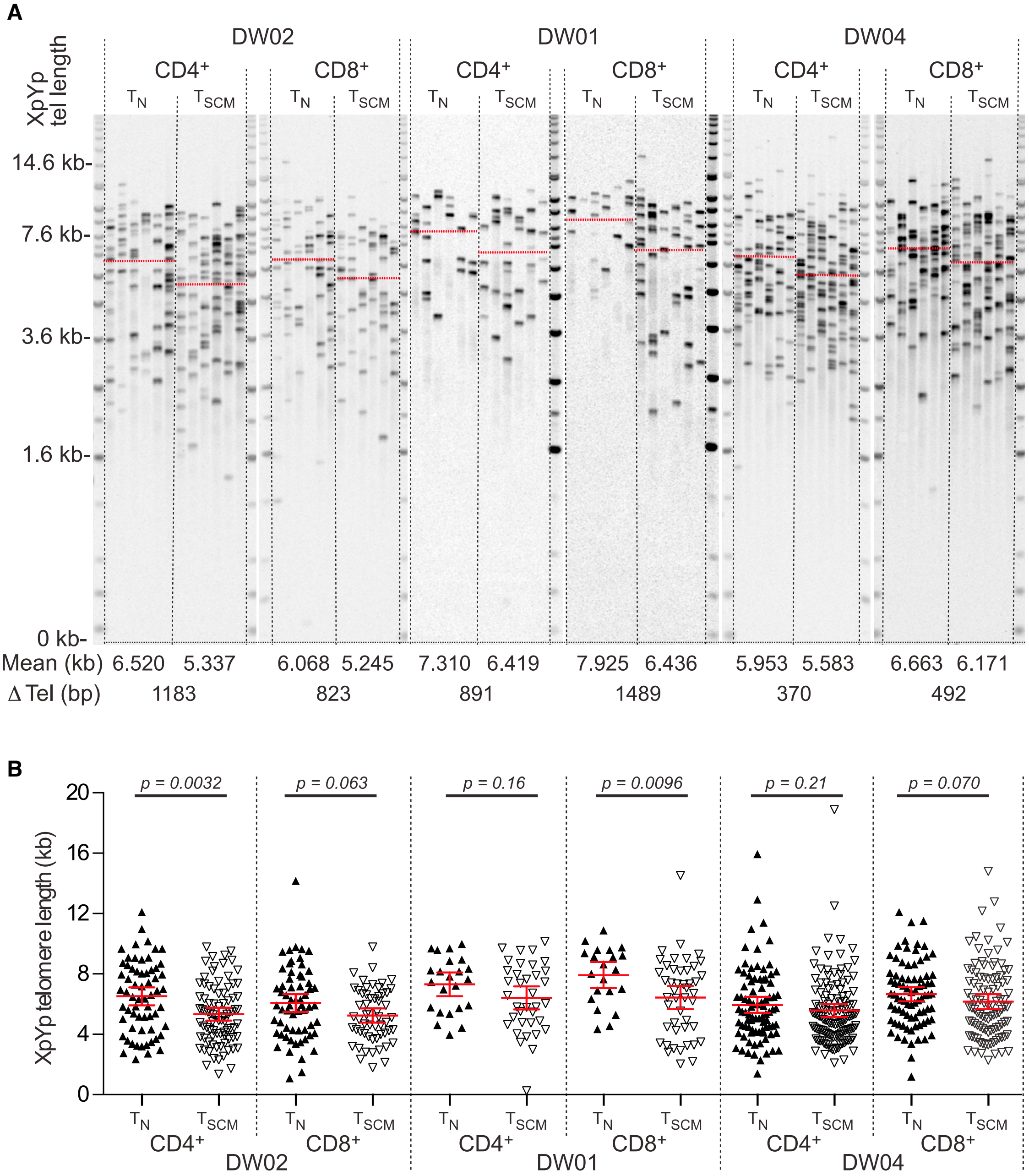

Figure 4. Telomere Lengths in Naive and Stem Cell-like Memory T Cells

(A) Representative single telomere length analysis data from subjects DW02 (elderly), DW01 (young adult), and DW04 (elderly). Single telomere length analysis was conducted at the XpYp telomere for $CD4^+$ and $CD8^+$ T_N and T_{SCM} cells. Mean values and telomere length differentials are shown (bottom).

(B) XpYp telomere length distributions as scatterplots. Significance was assessed using a two-tailed Mann-Whitney test.

S2A). Individual telomere lengths were distributed around a significantly lower mean in the T_{SCM} population compared with the T_N population ($CD4^+$ T cells, p = 0.0002; $CD8^+$ T cells, p = 0.0007; two-tailed Mann-Whitney p values pooled by Fisher's method) (Figures 4B and S2B). Moreover, T_{SCM} cells displayed higher levels of telomerase activity than either T_N or other memory T cells (Figure S2C). In the absence of telomerase activity, telomeres erode by 90 bp each time a population doubles in size (Baird et al., 2003). The telomere length differentials between T_{SCM} and T_N cells ranged from 370 to 1,489 bp (mean, 787 bp; Figures 4A, 4B, S2A, and S2B), equivalent to a maximum of almost 17 doublings at the population level. However, the true proliferative disparity will be substantially larger because telomerase markedly slows the rate of telomere

erosion. These data are again indicative of considerable turnover within the T_{SCM} compartment and further suggest a biological requirement for self-maintenance.

It remains unclear whether the T cell differentiation pathway is linear or bifurcated, with the latter model proposing that a single T_N cell gives rise to both a short-lived effector and a long-lived memory T cell (Arsenio et al., 2015; Flossdorf et al., 2015). There is some evidence for asymmetric division within the T_N pool (Chang et al., 2007; Arsenio et al., 2014), whereas other reports ascribe stemness to the T_{CM} pool (Graef et al., 2014). Irrespective of this ongoing debate, T_{SCM} cells are ideally equipped to amplify and preserve clonotypically encoded immunological memories (Gattinoni et al., 2011). In simian immunodeficiency virus-infected macaques, antigen-specific T_{SCM} cells display a 10-fold greater capacity to survive compared with T_{CM} cells following the loss of cognate antigen (Lugli et al., 2013). Similarly, vaccine-induced T_{SCM} cells can persist for decades with a naive-like profile (Fuertes Marraco et al., 2015). The T_{SCM} compartment is also preserved in HIV-infected individuals on long-term antiretroviral therapy (Vigano et al., 2015), despite the presence of a latent viral reservoir in the $CD4^+$ lineage (Jaafoura et al., 2014; Buzon et al., 2014). Further evidence attests to the proliferative capacity of T_{SCM} cells. In humans, the administration of cyclophosphamide after allogeneic bone marrow transplantation eradicates T_{SCM} cells, but leaves the T_N compartment largely intact (Roberto et al., 2015). Moreover, immune reconstitution is preferentially driven by T_{SCM} cells, at least in mice (Gattinoni et al., 2011). It therefore seems likely that the rapid turnover of T_{SCM} cells at the whole-population level reflects a composite of kinetically distinct subsets, potentially dissociated by transcriptional integration of variable antigenic stimuli and other immune activation signals (Cartwright et al., 2014; Lugli et al., 2013; Roychoudhuri et al., 2016). The data presented here are consistent with such divergent outcomes and suggest that nascent immunological memory is encapsulated within fluid cellular networks.

EXPERIMENTAL PROCEDURES

Human Samples

Seven healthy adults participated in the labeling study. Recruitment was stratified to include both young (aged 29–47 years) and elderly (aged 64–83 years) subjects, all of whom tested seropositive for cytomegalovirus and seronegative for HIV. Further peripheral blood samples were obtained from healthy adult volunteers. Approval was granted by the Cardiff University School of Medicine and London-Chelsea Research Ethics Committees. All studies were conducted according to the principles of the Declaration of Helsinki.

In Vivo Labeling

Study participants ingested small doses of 70% deuterated water (^2H_2O) over a 7-week period (50 ml three times daily for 1 week, then twice daily thereafter). Saliva samples were collected weekly for evaluation of body water labeling rates. Peripheral blood was collected at baseline and then at weeks 1, 3, 5, 7, 8, 10, 14, and 18. In one case (DW01), two further samples were collected (weeks 21 and 32).

Flow Cytometry and Cell Sorting

Peripheral blood mononuclear cells were isolated using standard density gradient centrifugation and stained with Live/Dead Fixable Aqua (Life Technologies), and anti-CD14-V500 and anti-CD19-V500 (BD Horizon) to exclude irrelevant signals from the analysis. The following monoclonal antibodies (mAbs) were used in further stains: (1) anti-CD3-H7APC, anti-CD28-APC, anti-CD45RA-PE, and anti-CD57-FITC (BD Pharmingen); (2) anti-CD4-Cy5.5PE and anti-CD27-QD605 (Life Technologies); (3) anti-CD45RO-ECD (Beckman Coulter); and (4) anti-CD8-BV711, anti-CD127-BV421, and anti-PD-1-BV421 (BioLegend). Naive ($CD27^{bright}CD45RO^-CCR7^+CD95^-$), stem cell-like memory ($CD27^{bright}CD45RO^-CCR7^+CD95^+$), transitional memory ($CD45RA^+CD45RO^+$), and memory ($CD45RA^-$) $CD4^+$ and $CD8^+$ T cells were sorted at >98% purity using a custom-modified FACSAria II flow cytometer (BD Biosciences). Intracellular expression of Ki67 was evaluated separately using an Alexa Fluor 647-conjugated mAb in conjunction with a Cytofix/Cytoperm Kit (BD Biosciences). Data were analyzed with FlowJo software, version 9.7.6 (Tree Star).

Measurement and Analysis of ^2H Enrichment in T Cell DNA

The stable isotope-based method for measuring T cell proliferation has been described previously (Hellerstein et al., 1999; McCune et al., 2000; Neese et al., 2001). Additional precautions and controls were incorporated to ensure the accurate quantification of ^2H enrichment in low-abundance samples (Busch et al., 2007). Briefly, DNA from sort-purified T cell subsets was released by boiling and hydrolyzed according to standard protocols. Deoxyribonucleosides were derivatized using pentafluorobenzyl hydroxylamine (Sigma-Aldrich). Gas chromatography/mass spectrometry (Agilent 5873/6980) was performed in negative chemical ionization mode using a DB-17 column (J&W Scientific; Agilent). The M+1/M+0 isotopomer ratio was monitored at mass-to-charge (*m/z*) 436/435. To normalize for body water enrichment, weekly saliva samples were analyzed for ^2H_2O content via calcium carbide-induced acetylene generation, monitoring at *m/z* 27/26 (Previs et al., 1996).

Single Telomere Length Analysis

DNA was extracted from 3,000 sort-purified T cells using a QIAmp DNA Micro Kit (QIAGEN). Single telomere length analysis was carried out at the XpYp telomere as described previously (Capper et al., 2007). Briefly, 1 μM of the Telorette-2 linker was added to purified genomic DNA in a final volume of 40 μL per sample. Multiple PCRs were performed for each test DNA in 10-μL volumes incorporating 250 pg of DNA and 0.5 μM of the telomere-adjacent and Teltail primers in 75 mM Tris-HCl pH 8.8, 20 mM $(NH_4)_2SO_4$, 0.01% Tween-20, and 1.5 mM $MgCl_2$, with 0.5 U of a 10:1 mixture of Taq (ABGene) and Pwo polymerase (Roche Molecular Biochemicals). The reactions were processed in a Tetrad2 Thermal Cycler (Bio-Rad). DNA fragments were resolved by 0.5% Tris-acetate-EDTA agarose gel electrophoresis and identified by Southern hybridization with a random-primed α-^{33}P-labeled (PerkinElmer) TTAGGG repeat probe, together with probes specific for the 1-kb (Stratagene) and 2.5-kb (Bio-Rad) molecular weight markers. Hybridized fragments were detected using a Typhoon FLA 9500 Phosphorimager (GE Healthcare). The molecular weights of the DNA fragments were calculated using a Phoretix 1D Quantifier (Nonlinear Dynamics).

Telomerase Activity

Sort-purified T cells were lyzed and assayed in two steps using a modified SYBR Green real-time quantitative telomerase repeat amplification protocol (Wege et al., 2003). Standard curves were obtained from serial dilutions of a 293T cell extract with known telomerase activity. Experimental telomerase activity was calculated with reference to 293T cells and expressed as relative telomerase activity (Ct_{293T}/Ct_{sample}).

Mathematical Modeling

Four mathematical models describing the relationship between T_N and T_{SCM} cells were constructed using ordinary differential equations and fitted to the labeling data in R. Two variations were also considered for each model: (1) proliferation was factored into the peripheral blood T_N pool; and (2) the precursor compartment was omitted for T_N cells. None of these variants yielded better predictions than the original models. The following equations were used to

describe the rate of change of the fraction of labeled DNA in the precursor and T_N compartments:

$$\dot{F}_A = r_A(cU - F_A)$$

$$\dot{F}_{TN} = (d_1 + \Delta)(F_A - F_{TN}),$$

where r_A represents the rate at which naive cells move from A to T_N, d_1 is the disappearance rate of T_N cells in the blood, Δ is the differentiation rate (associated with proliferation in models 1 and 2) of T_N into T_{SCM} cells, c is the amplification factor for enrichment, and U is the function describing labeling and delabeling in saliva. The following equations were used for the T_{SCM} pool:

$$\text{Model 1}: \ \dot{F}_{TSCM} = \frac{\Delta T_N}{T_{SCM}}(cU + F_N) + pcU - \left(\frac{2\Delta T_N}{T_{SCM}} + p\right)F_{TSCM}$$

$$\text{Model 2}: \ \dot{F}_{TSCM} = \frac{\Delta T_N}{T_{SCM}}(cU + F_N - 2F_{TSCM})$$

$$\text{Model 3}: \ \dot{F}_{TSCM} = \frac{\Delta T_N}{T_{SCM}}F_N + pcU - \left(\frac{\Delta T_N}{T_{SCM}} + p\right)F_{TSCM}$$

$$\text{Model 4}: \ \dot{F}_{TSCM} = \frac{\Delta T_N}{T_{SCM}}(F_N - F_{TSCM}),$$

where p is the rate of proliferation within the T_{SCM} pool and T_N/T_{SCM} is the ratio of the sizes of the T_N and T_{SCM} pools measured experimentally. Model fits were compared using the corrected Akaike information criterion (Burnham and Anderson, 2002).

Statistical Analysis

Telomere lengths between the T_N and T_{SCM} populations were compared using a two-tailed Mann-Whitney test. The p values were pooled using Fisher's method.

SUPPLEMENTAL INFORMATION

Supplemental Information includes two figures and can be found with this article online at http://dx.doi.org/10.1016/j.celrep.2016.11.037.

AUTHOR CONTRIBUTIONS

R.A., L.R., K.L.M., R.E.J., T.F., Y.Z., and K.L. carried out experiments. R.A., L.R., R.E.J., V.A., D.M.B., D.C.M., and K.L. analyzed data. P.C.d.A., L.B., M.E., and B.A. modeled data. D.A.P., D.C.M., and K.L. designed experiments. P.C.d.A., L.B., V.A., D.M.B., and B.A. edited the manuscript. D.A.P., D.C.M., and K.L. wrote the manuscript.

ACKNOWLEDGMENTS

This work was funded by the Wellcome Trust (Grant 093053/Z/10/Z), the Medical Research Council (Grant G1001052), and Cancer Research UK (Grant C17199/A18246). B.A. and D.A.P. are Wellcome Trust Investigators. The authors extend their profound thanks to all study participants.

Received: June 27, 2016
Revised: September 23, 2016
Accepted: November 10, 2016
Published: December 13, 2016

REFERENCES

Arsenio, J., Kakaradov, B., Metz, P.J., Kim, S.H., Yeo, G.W., and Chang, J.T. (2014). Early specification of CD8+ T lymphocyte fates during adaptive immunity revealed by single-cell gene-expression analyses. Nat. Immunol. *15*, 365–372.

Arsenio, J., Kakaradov, B., Metz, P.J., Yeo, G.W., and Chang, J.T. (2015). Reply to: "CD8+ T cell diversification by asymmetric cell division". Nat. Immunol. *16*, 893–894.

Baird, D.M., Rowson, J., Wynford-Thomas, D., and Kipling, D. (2003). Extensive allelic variation and ultrashort telomeres in senescent human cells. Nat. Genet. *33*, 203–207.

Burnham, K.P., and Anderson, D.R. (2002). Model Selection and Multimodel Inference: A Practical Information-Theoretic Approach (Springer).

Busch, R., Neese, R.A., Awada, M., Hayes, G.M., and Hellerstein, M.K. (2007). Measurement of cell proliferation by heavy water labeling. Nat. Protoc. *2*, 3045–3057.

Buzon, M.J., Sun, H., Li, C., Shaw, A., Seiss, K., Ouyang, Z., Martin-Gayo, E., Leng, J., Henrich, T.J., Li, J.Z., et al. (2014). HIV-1 persistence in CD4+ T cells with stem cell-like properties. Nat. Med. *20*, 139–142.

Capper, R., Britt-Compton, B., Tankimanova, M., Rowson, J., Letsolo, B., Man, S., Haughton, M., and Baird, D.M. (2007). The nature of telomere fusion and a definition of the critical telomere length in human cells. Genes Dev. *21*, 2495–2508.

Cartwright, E.K., McGary, C.S., Cervasi, B., Micci, L., Lawson, B., Elliott, S.T., Collman, R.G., Bosinger, S.E., Paiardini, M., Vanderford, T.H., et al. (2014). Divergent CD4+ T memory stem cell dynamics in pathogenic and nonpathogenic simian immunodeficiency virus infections. J. Immunol. *192*, 4666–4673.

Chang, J.T., Palanivel, V.R., Kinjyo, I., Schambach, F., Intlekofer, A.M., Banerjee, A., Longworth, S.A., Vinup, K.E., Mrass, P., Oliaro, J., et al. (2007). Asymmetric T lymphocyte division in the initiation of adaptive immune responses. Science *315*, 1687–1691.

Chang, J.T., Wherry, E.J., and Goldrath, A.W. (2014). Molecular regulation of effector and memory T cell differentiation. Nat. Immunol. *15*, 1104–1115.

Di Benedetto, S., Derhovanessian, E., Steinhagen-Thiessen, E., Goldeck, D., Müller, L., and Pawelec, G. (2015). Impact of age, sex and CMV-infection on peripheral T cell phenotypes: results from the Berlin BASE-II Study. Biogerontology *16*, 631–643.

Farber, D.L., Yudanin, N.A., and Restifo, N.P. (2014). Human memory T cells: generation, compartmentalization and homeostasis. Nat. Rev. Immunol. *14*, 24–35.

Flossdorf, M., Rössler, J., Buchholz, V.R., Busch, D.H., and Höfer, T. (2015). CD8+ T cell diversification by asymmetric cell division. Nat. Immunol. *16*, 891–893.

Fuertes Marraco, S.A., Soneson, C., Cagnon, L., Gannon, P.O., Allard, M., Abed Maillard, S., Montandon, N., Rufer, N., Waldvogel, S., Delorenzi, M., and Speiser, D.E. (2015). Long-lasting stem cell-like memory CD8+ T cells with a naïve-like profile upon yellow fever vaccination. Sci. Transl. Med. *7*, 282ra48.

Gattinoni, L., Lugli, E., Ji, Y., Pos, Z., Paulos, C.M., Quigley, M.F., Almeida, J.R., Gostick, E., Yu, Z., Carpenito, C., et al. (2011). A human memory T cell subset with stem cell-like properties. Nat. Med. *17*, 1290–1297.

Graef, P., Buchholz, V.R., Stemberger, C., Flossdorf, M., Henkel, L., Schiemann, M., Drexler, I., Höfer, T., Riddell, S.R., and Busch, D.H. (2014). Serial transfer of single-cell-derived immunocompetence reveals stemness of CD8+ central memory T cells. Immunity *41*, 116–126.

Hellerstein, M., Hanley, M.B., Cesar, D., Siler, S., Papageorgopoulos, C., Wieder, E., Schmidt, D., Hoh, R., Neese, R., Macallan, D., et al. (1999). Directly measured kinetics of circulating T lymphocytes in normal and HIV-1-infected humans. Nat. Med. *5*, 83–89.

Hellerstein, M.K., Hoh, R.A., Hanley, M.B., Cesar, D., Lee, D., Neese, R.A., and McCune, J.M. (2003). Subpopulations of long-lived and short-lived T cells in advanced HIV-1 infection. J. Clin. Invest. *112*, 956–966.

Jaafoura, S., de Goër de Herve, M.G., Hernandez-Vargas, E.A., Hendel-Chavez, H., Abdoh, M., Mateo, M.C., Krzysiek, R., Merad, M., Seng, R., Tardieu, M., et al. (2014). Progressive contraction of the latent HIV reservoir around a core of less-differentiated CD4+ memory T Cells. Nat. Commun. *5*, 5407.

Ladell, K., Hellerstein, M.K., Cesar, D., Busch, R., Boban, D., and McCune, J.M. (2008). Central memory CD8+ T cells appear to have a shorter lifespan and reduced abundance as a function of HIV disease progression. J. Immunol. *180*, 7907–7918.

Ladell, K., Hazenberg, M.D., Fitch, M., Emson, C., McEvoy-Hein Asgarian, B.K., Mold, J.E., Miller, C., Busch, R., Price, D.A., Hellerstein, M.K., and McCune, J.M. (2015). Continuous antigenic stimulation of DO11.10 TCR transgenic mice in the presence or absence of IL-1β: possible implications for mechanisms of T cell depletion in HIV disease. J. Immunol. *195*, 4096–4105.

Lugli, E., Dominguez, M.H., Gattinoni, L., Chattopadhyay, P.K., Bolton, D.L., Song, K., Klatt, N.R., Brenchley, J.M., Vaccari, M., Gostick, E., et al. (2013). Superior T memory stem cell persistence supports long-lived T cell memory. J. Clin. Invest. *123*, 594–599.

McCune, J.M., Hanley, M.B., Cesar, D., Halvorsen, R., Hoh, R., Schmidt, D., Wieder, E., Deeks, S., Siler, S., Neese, R., and Hellerstein, M. (2000). Factors influencing T-cell turnover in HIV-1-seropositive patients. J. Clin. Invest. *105*, R1–R8.

Neese, R.A., Siler, S.Q., Cesar, D., Antelo, F., Lee, D., Misell, L., Patel, K., Tehrani, S., Shah, P., and Hellerstein, M.K. (2001). Advances in the stable isotope-mass spectrometric measurement of DNA synthesis and cell proliferation. Anal. Biochem. *298*, 189–195.

Neese, R.A., Misell, L.M., Turner, S., Chu, A., Kim, J., Cesar, D., Hoh, R., Antelo, F., Strawford, A., McCune, J.M., et al. (2002). Measurement in vivo of proliferation rates of slow turnover cells by ^2H_2O labeling of the deoxyribose moiety of DNA. Proc. Natl. Acad. Sci. USA *99*, 15345–15350.

Previs, S.F., Hazey, J.W., Diraison, F., Beylot, M., David, F., and Brunengraber, H. (1996). Assay of the deuterium enrichment of water via acetylene. J. Mass Spectrom. *31*, 639–642.

Roberto, A., Castagna, L., Zanon, V., Bramanti, S., Crocchiolo, R., McLaren, J.E., Gandolfi, S., Tentorio, P., Sarina, B., Timofeeva, I., et al. (2015). Role of naive-derived T memory stem cells in T-cell reconstitution following allogeneic transplantation. Blood *125*, 2855–2864.

Roychoudhuri, R., Clever, D., Li, P., Wakabayashi, Y., Quinn, K.M., Klebanoff, C.A., Ji, Y., Sukumar, M., Eil, R.L., Yu, Z., et al. (2016). BACH2 regulates CD8+ T cell differentiation by controlling access of AP-1 factors to enhancers. Nat. Immunol. *17*, 851–860.

Scholzen, T., and Gerdes, J. (2000). The Ki-67 protein: from the known and the unknown. J. Cell. Physiol. *182*, 311–322.

Vigano, S., Negron, J., Ouyang, Z., Rosenberg, E.S., Walker, B.D., Lichterfeld, M., and Yu, X.G. (2015). Prolonged antiretroviral therapy preserves HIV-1-specific CD8 T cells with stem cell-like properties. J. Virol. *89*, 7829–7840.

Vrisekoop, N., den Braber, I., de Boer, A.B., Ruiter, A.F., Ackermans, M.T., van der Crabben, S.N., Schrijver, E.H., Spierenburg, G., Sauerwein, H.P., Hazenberg, M.D., et al. (2008). Sparse production but preferential incorporation of recently produced naive T cells in the human peripheral pool. Proc. Natl. Acad. Sci. USA *105*, 6115–6120.

Wege, H., Chui, M.S., Le, H.T., Tran, J.M., and Zern, M.A. (2003). SYBR Green real-time telomeric repeat amplification protocol for the rapid quantification of telomerase activity. Nucleic Acids Res. *31*, E3-3.

Cell Reports, Volume *17*

Supplemental Information

Human Stem Cell-like Memory T Cells

Are Maintained in a State of Dynamic Flux

Raya Ahmed, Laureline Roger, Pedro Costa del Amo, Kelly L. Miners, Rhiannon E. Jones, Lies Boelen, Tinhinane Fali, Marjet Elemans, Yan Zhang, Victor Appay, Duncan M. Baird, Becca Asquith, David A. Price, Derek C. Macallan, and Kristin Ladell

Supplemental Figure 1 (related to Figure 1):

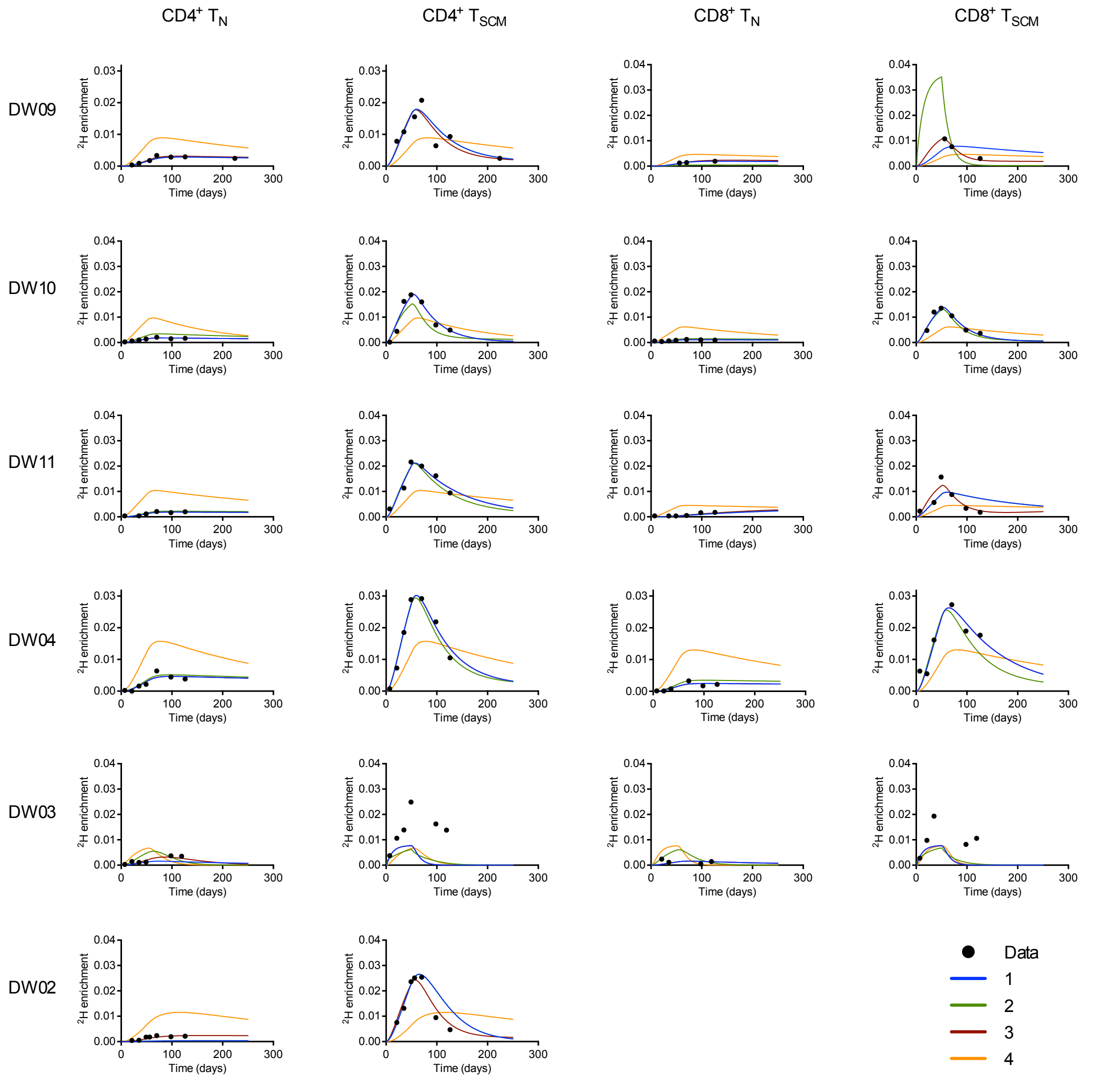

Supplemental Figure 1. Label incorporation in naïve and stem cell-like memory T cells. Experimental labeling data (black filled circles) and modeled curve fits for subjects DW09, DW10 and DW11 (young adults), and DW04, DW03 and DW02 (elderly).

Supplemental Figure 2 (related to Figure 4):

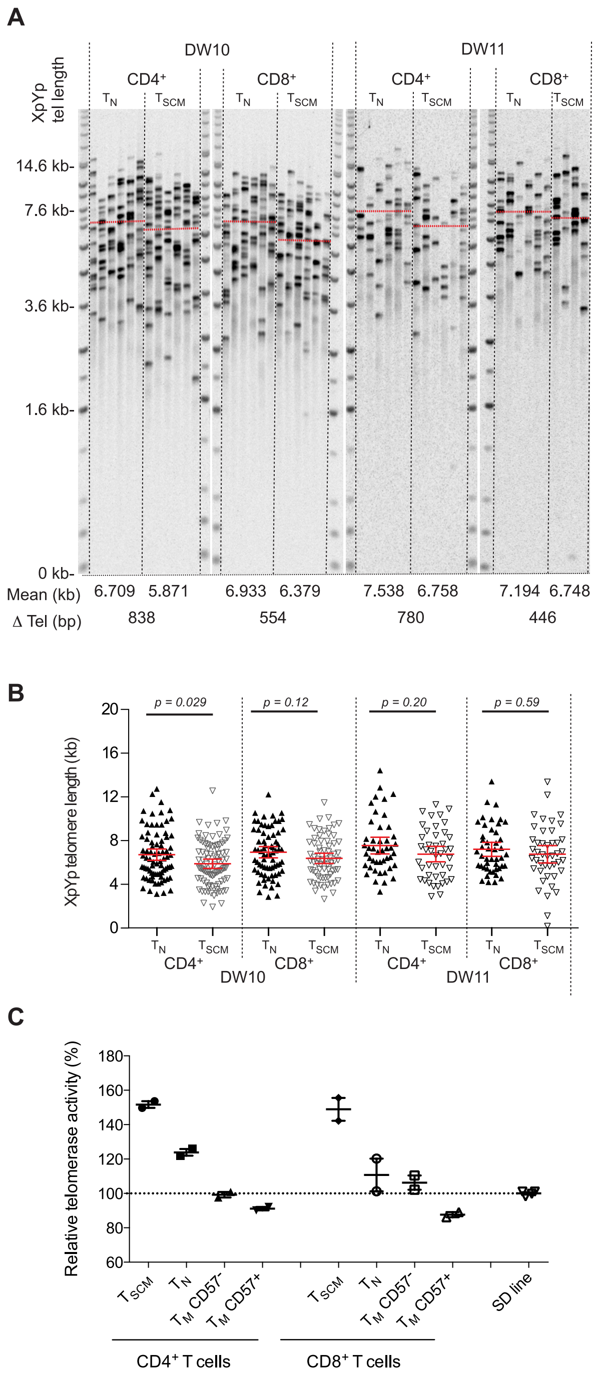

Supplemental Figure 2. Telomere lengths and telomerase activity in naïve and stem cell-like memory T cells. (A) Representative STELA data from subjects DW10 and DW11 (young adults). STELA was conducted at the XpYp telomere for CD4+ and CD8+ T_N and T_{SCM} cells. Mean values and telomere length differentials are shown (bottom). (B) XpYp telomere length distributions as scatter plots. Significance was

assessed using a two-tailed Mann-Whitney test. (C) Relative telomerase activity for $CD4^+$ and $CD8^+$ T_N, T_{SCM} and memory T (T_M) cells from subjects DW02 and DW04 (elderly). T_M cells are segregated as $CD57^-$ (less differentiated) and $CD57^+$ (more differentiated). Horizontal bars represent mean values with standard errors.
